# Alternating electric fields transform the intricate network of tumour vasculature into orderly parallel capillaries and enhance the anti‐angiogenesis effect of bevacizumab

**DOI:** 10.1111/cpr.13734

**Published:** 2024-08-19

**Authors:** Lin Shen, Shuai Li, Yalin Wang, Yi Yin, Yiting Liu, Yunlei Zhang, Xuesheng Zheng

**Affiliations:** ^1^ Department of Neurosurgery, XinHua Hospital Shanghai China; ^2^ Department of Neurosurgery The Affiliated Jiangning Hospital of Nanjing Medical University Nanjing China; ^3^ School of electronic information and electrical engineering Shanghai Jiao Tong University Shanghai China; ^4^ Department of Respiratory and Critical Care Medicine The Affiliated Jiangning Hospital of Nanjing Medical University, Nanjing Medical University Nanjing China; ^5^ The Key Laboratory of Clinical and Medical Engineering, School of Biomedical Engineering and Informatics Nanjing Medical University Nanjing China

## Abstract

The search for effective strategies to target tumour angiogenesis remains a critical goal of cancer research. We present a pioneering approach using alternating electric fields to inhibit tumour angiogenesis and enhance the therapeutic efficacy of bevacizumab. Chicken chorioallantoic membrane, cell viability and in vitro endothelial tube formation assays revealed that electric fields with a frequency of 1000 kHz and an electric intensity of 0.6 V/cm inhibited the growth of vascular endothelial cells and suppressed tumour‐induced angiogenesis. In an animal U87MG glioma model, 1000 kHz electric fields inhibited tumour angiogenesis and suppressed tumour growth. As demonstrated by 3D vessel analysis, tumour vasculature in the control group was a stout, interwoven network. However, electric fields transformed it into slim, parallel capillaries that were strictly perpendicular to the electric field direction. This architectural transformation was accompanied by apoptosis of vascular endothelial cells and a notable reduction in tumour vessel number. Additionally, we found that the anti‐angiogenesis and tumour‐suppression effects of electric fields synergised with bevacizumab. The anti‐angiogenic mechanisms of electric fields include disrupting spindle formation during endothelial cell division and downregulating environmental angiogenesis‐related cytokines, such as interleukin‐6, CXCL‐1, 2, 3, 5 and 8, and matrix metalloproteinases. In summary, our findings demonstrate the potential of alternating electric fields (AEFs) as a therapeutic modality to impede angiogenesis and restrain cancer growth.

## Graphical abstract

The application of 1000 kHz alternating electric fields effectively suppresses tumour vasculature, transforming it into orderly, parallel straight capillaries. This not only halts the growth of xenograft tumours but also synergistically enhances the therapeutic effects of bevacizumab, offering a promising approach in cancer treatment.

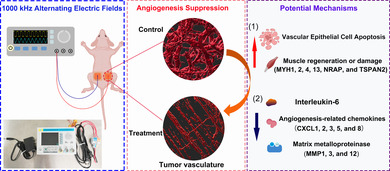

## 1 INTRODUCTION

Tumour treating fields (TTFields) have emerged as a promising modality in cancer therapy, utilising alternating electric fields of moderate frequency (100–300 kHz) and low intensity (1–3 V/cm) to inhibit tumour growth.^1^ Through their selective influence on proliferating cells, TTFields exhibit forces upon highly polar intracellular components, including crucial molecules such as tubulin and septins.^2^ This interference causes abnormal microtubule polymerisation during spindle formation, leading to irregular cleavage furrow and subsequent disruption of chromosome, macromolecule and organelle segregation during mitosis.^1^, ^3^ Consequently, aberrant and prolonged division often leads to apoptosis, accompanied by cell membrane blebbing and eventual rupture.^4^, ^5^ However, it's reported that electric fields at frequencies of 500 kHz or above have little measureable effect on tumour cells.^2^

Recent studies have revealed the potential of TTFields to disrupt cellular signalling pathways, including nuclear factor kappa‐B (NF‐κB), stimulator of interferon genes (STING), adenosine 5′‐monophosphate‐activated protein kinase (AMPK), signal transducer and activator of transcription 3 (STAT3) and c‐Jun N‐terminal kinase (JNK), which govern cellular responses to environmental cues such as inflammation, cellular growth and apoptosis.^5^, ^6^, ^7^ TTFields also affect cancer cell metabolism, including glycolysis, hence constraining cell proliferation.^8^ Another facet of TTFields' influence is the downregulation of tight junction proteins among neurovascular endothelial cells, which enhances blood–brain barrier permeability and facilitates improved drug penetration into brain tumours.^9^ Additionally, TTFields increase the exposure of calmodulin on tumour cell membranes, promoting enhanced dendritic cell maturation and elevated recruitment of immune cells, thus enhancing the efficacy of programmed cell death‐1 (PD‐1)‐targeted immunotherapy.^10^ Moreover, TTFields inhibit tumour cell migration and invasion by downregulating diverse factors such as vascular endothelial growth factor (VEGF), hypoxia inducible factor‐1 alpha (HIF1α), matrix metalloproteinases 2 and 9, vimentin and E‐cadherin.^11^, ^12^ The mechanisms underlying the effects of alternating electric fields on tumour growth have garnered significant scientific attention, as elucidating their nuances is essential for optimising their integration into cancer therapy.

Angiogenesis, the formation of new blood vessels, plays a crucial role in the progression of malignant tumours. The newly formed vessels provide a conduit for the delivery of oxygen and nutrients to tumour cells, enabling their growth and proliferation. Additionally, they serve as a pathway for tumour cells to enter the systemic circulation and metastasise to other parts of the body.^13^ Inhibition of tumour angiogenesis stands as a promising therapeutic approach in cancer treatment, as evidenced by the success of bevacizumab, a humanised anti‐VEGF monoclonal antibody.^14^ Tumour angiogenesis is primarily driven by active endothelial cell proliferation. In contrast, endothelial cells in normal adult tissues are generally quiescent, with few exceptions, such as the uterus.^15^ Because alternating electric fields selectively target proliferating cells and leave quiescent cells unharmed,^1^ we hypothesise that alternating electric fields of specific parameters could be used to suppress tumour angiogenesis by affecting proliferating endothelial cells. This study is designed to test this hypothesis. Additionally, we examined whether electric fields work synergistically with bevacizumab.

## 2 METHODS

### 2.1 Study design

The objective of this investigation was to devise an innovative approach that harnesses alternating electric fields with the aim of selectively diminishing angiogenesis. The sample size was determined in adherence to established protocols. All collected data, including potential outliers, were duly incorporated. For each experimental condition, a minimum of three mice were randomly allocated. To ensure objectivity, immunohistochemistry, transcriptomic analysis and fluorescent imaging procedures were performed under blinded conditions.

### 2.2 Cell culture and alternating electric fields

The Human Umbilical Vein Endothelial Cells (HUVECs), U87MG glioblastoma cell line and MCF‐7 breast cancer cell line were sourced from the Cell Bank of the Chinese Academy of Sciences in Shanghai, China. The cancer cells were propagated in Dulbecco's modified Eagle's medium (DMEM) (Gibco, USA), which was supplemented with 10% (v/v) foetal bovine serum (FBS) (Gibco). This cultivation was carried out at 37°C within a humidified incubator possessing a 5% CO_2_ atmosphere. Distinctively, the HUVECs underwent cultivation in specialised endothelial cell medium (ScienCell, USA), which was supplemented with 10% FBS, 0.05 mg/mL of endothelial cell growth supplement, and 1% (v/v) penicillin/streptomycin. This specialised cell culture was conducted at 37°C within a dedicated cell culture facility tailored for TTFields application, which is equipped with an alternating current generator, a customised culture platform, and culture flasks (Shanghai, China. http://www.ttfieldschina.com). The alternating current generator is endowed with the capacity to deliver sine waves characterised by a maximum amplitude (U) of 100 V peak to peak (vpp) and frequencies spanning up to 2 MHz. As delineated in Figure S1, the culture platform was integrated within the confines of a cell incubator's culture chamber. It was linked to the alternating current generator via copper wires that traversed a designated cable port. The culture platform featured an assemblage of copper ball connectors. Notably, these connectors firmly established contact with the plate electrodes affixed to the exterior wall of the culture flask. In consequence, upon flask insertion, the ball connectors seamlessly interfaced with the plate electrodes, facilitating the conduction of alternating current. The flask wall served as an insulating layer. The culture flask is cuboid‐shaped with the bottom length of 1.8 cm (d1) and the wall thickness of 0.06 cm (d2). The culture flask wall has a relative dielectric constant (ε2) of 4.1 at 37°C. The cell culture medium and animal plasma have a relative dielectric constant (ε1) of approximately 1000, as evidenced by literatures.^4^ With the parameters mentioned above (U, d1, ε1, d2, ε2), the electric field intensity (E1) in the culture medium was calculated using the online U‐Shaped System Electric Field Intensity (USSEFI) tool (http://www.ttfieldschina.com/doku.php?id=ussefi).

### 2.3 Cytotoxicity assay and confocal imaging

The HUVECs were subjected to alternating electric fields with varying frequencies (200, 500, 1000 and 1500 kHz) or electric field intensities (0.3, 0.6, 0.9 and 1.2 V/cm) for a duration of 48 h. Subsequent to this exposure, the viability of the HUVECs was meticulously gauged using the Cell Counting Kit 8, in accordance with the recommended protocol from Dojindo, Japan. The optical density (OD) measurements were registered at 450 nm, utilising a microplate reader sourced from BioTek, Winooski, VT, USA. Concurrently, a parallel cohort of cells underwent treatment with Vincristine, administered at a concentration of 5 μM, and this intervention was sustained over the course of 48 h. Following this treatment, the cells were promptly fixed with ice‐cold 100% methanol for a duration of 5 min while maintained on ice. In sequence, the cellular permeabilisation process was carried out by 5‐min incubation in a 0.1% Triton X‐100 solution. Then, the permeabilised cells were immersed into a specialised blocking buffer (Beyotime, Shanghai, China) for 1 h. Subsequently, the cells underwent three consecutive washes with Phosphate Buffered Saline (PBS). The cells were incubated in the Alexa Fluor® 647 labelling anti‐Histone H3 (phospho S10) antibodies with a 1: 100 dilution and the Alexa Fluor® 488 labelling anti‐α‐tubulin at a 1/250 dilution (Abcam, USA) overnight at 4°C. Nuclear DNA was subsequently labelled using 4',6‐Diamidino‐2‐phenylindole (DAPI). Images of cellular staining were captured using an inverted confocal laser scanning microscope (LSM 710, Carl Zeiss, Germany).

### 2.4 Chicken chorioallantoic membrane (CAM) assay

CAM assay was carried out to screen for an optimal frequency for inhibiting tumour‐stimulated angiogenesis. A hole with a diameter of 0.6 cm was made in the shell of each egg with a 10‐day‐old chicken embryo (Shanghai Veterinary Research Institute, Shanghai, China). The hole is located above the egg air chamber. A small piece of the dermic sheet on the floor of the air chamber was removed, and filter paper with a diameter of 0.5 cm was placed there. Then MCF‐7 or U87MG tumour cells (50 μL, 1 × 10^6^) were added onto the centre of the paper. Afterwards the hole was sealed with sterile adhesive tape, and the eggs were incubated at 37°C under 75% humidity for 48 h. The CAMs were fixed by 1:1 methanol/acetone solution for 30 min, cut and harvested. Photos of each CAM were taken using a digital camera (Nikon, Tokyo Metropolis, Japan). To apply alternating electric fields on the CAM, a pair of copper plate electrodes with a side length of 2 cm were stuck on the egg surface with conductive adhesive, and then the electrodes were connected with the alternating current generator (Figure S1). Alternating electric fields were applied at the following frequencies: 30, 50, 100, 200, 300, 500, 800, 1000 and 1500 kHz for the CAM study with MCF‐7 as the stimulator, and 200, 500, 1000 and 1500 kHz for the assay involving U87MG as the stimulator. Distance between the electrodes on the eggs was 3.92 ± 0.25 cm (d1), and the relative dielectric constant was 1000 (ε1).^4^ The egg shell served as the insulating layer, with a relative dielectric constant (ε2) of 6.1, and its thickness (d2) was 0.035 ± 0.002 cm. The voltage was set at 9.2 Vpp, resulting in an electric field intensity of approximately 0.6 V/cm, as calculated using the USSEFI tool. The choice of MCF‐7 and U87MG cell lines as stimuli in the CAM assay was based on their stable proangiogenic effect,^16^, ^17^, ^18^ thus allowing us to focus solely on assessing the specific effects of AEF on angiogenesis.

### 2.5 HUVECs tube formation assay

Firstly, 80 μL Matrigel (BD Biosciences, Massachusetts, USA) was added into cell flask and incubated for 1 h at 37°C, and then 1 mL of 1 × 10^4^/100 μL HUVECs were added to each flask and incubated at 37°C in an incubator with 5% CO_2_ for 3 h, when alternating electric fields (200, 500, 1000 and 1500 KHz) at an electric field intensity of 0.6 V/cm were applied to the cells. After 24 h incubation, the tube formation images were captured under a microscope.

### 2.6 Animal xenograft tumour model and alternating electric fields treatment

The animal study was approved by the Animal Ethics Committee of JiangNing Hospital. Female Nude BALB/c mice, aged between 6 and 8 weeks, were selected for the study. These mice were subcutaneously injected with a 100 μL suspension of U87MG cells, with each injection containing 1 X 10^6^ cells. The injection site was localised to the buttock. Tumour volumes were measured using Vernier callipers and calculated using the formula 0.52 × (length × width × width). Once the xenograft tumours had achieved discernible visibility, typically ranging between 100 and 200 mm^3^, the experimental interventions commenced. A pair of insulated copper plate electrodes, characterised by a square shape and a side length of 1 cm, were affixed to the tumour surface of all mice enrolled in the experiments. The secure adhesion of these electrodes was facilitated using adhesive tape. The insulating layer, a thickness (d2) of 0.005 cm, featured a relative dielectric constant (ε2) of 3.0. The distance maintained between the electrodes was calibrated at 1.54 ± 0.18 cm (d1), while the relative dielectric constant (ε1) was approximately 1000.^4^ For the purpose of accurate quantification, the electric field intensity was calculated employing the U‐Shaped System Electric Field Intensity (USSEFI) tool. The experimental trajectory encompassed two distinct phases. In the initial phase, aimed at observing the structural characteristics and distribution of tumour vasculature, the tumour‐bearing mice were subjected to treatment either by AEFs or bevacizumab. The latter intervention involved i.t. administering bevacizumab at a dosage of 10 μg per gram of mouse, conducted twice a week. Mice with copper plate electrodes affixed but without electric fields served as the control group. In a subsequent phase, the objective is to explore the anti‐angiogenic effects of AEFs and its potential synergy with bevacizumab. Accordingly, electric fields were administered over a span of 2 weeks, while bevacizumab was i.v. administered at a dose of 10 μg per gram of mouse, carried out four times within the stipulated treatment timeframe. After the treatment, the mice were sacrificed under deep anaesthesia and tumours were excised for multifaceted analyses, including weight measurement, immunohistochemistry, TUNEL assay, and transcriptomics analysis. Each cage accommodated three mice and underwent inspection at five‐hour intervals. This evaluation aimed to verify the secure placement of copper plate electrodes at the tumour sites of the mice while ensuring that wires remained free from entanglement. To mitigate potential wire damage by the mice, a protective cloth tape was applied. It is worth noting that the flexibility of the wire allowed unencumbered movement for the mice, facilitating their access to food and water. The duration of the above‐mentioned animal experiments is illustrated in Figures 2B and 4A.

### 2.7 In vivo two‐photon microscopic imaging (ghost imaging)

For in vivo two‐photon microscopic imaging of tumours, a tumour window installation surgery was conducted on the animals. Prior to the installation procedure, the mice were subjected to isoflurane inhalation anaesthesia and were securely positioned within a stereotaxic frame to ensure precise immobilisation. The execution of the tumour window entailed the creation of an aperture measuring 3 mm in diameter on the tumour's surface. To facilitate this, a customised chamber frame was positioned around the exposed tumour area and securely affixed using cyanoacrylic glue. Following this preparatory phase, the mice received an intravenous injection of 100 μL Texas red‐conjugated dextran (40 kDa) at a concentration of 10 mg/mL (Invitrogen, USA). Following a brief interval of 5 min, the tumours were subjected to two‐photon microscopy, realised using a specialised system (FLUOVIEW FVMPE‐RS, OLYMPUS, Japan). The microscopy process entailed the immersion of the microscope's lens in water, maintained within the confines of the customised chamber frame. This configuration ensured an optimal imaging environment while providing the requisite stability for capturing high‐resolution images. The ghost imaging was processed using Imaris 9 software (OXFORD Instruments) to generate the simulation diagrams following the instructions.

### 2.8 Immunohistochemistry and TUNEL assay

Tumour specimens, as well as normal organs, including the lung, heart, kidney, spleen and liver, were excised and immersed in a 4% paraformaldehyde solution overnight. The samples were subjected to a processing sequence in accordance with established protocols for fluorescent immunohistochemistry or immunohistochemistry. In particular, sections from the tumour samples were subjected to an overnight incubation with anti‐CD31 antibody conjugated with Alexa Fluor® 555 (diluted at 1:100; sourced from ABCAM, USA) and α‐Tubulin antibody conjugated with Alexa Fluor® 488 (diluted at 1:200; sourced from ABCAM, USA) held at a temperature of 4°C. Following a rigorous triple rinse using PBS, the samples were subjected to staining with DAPI (0.5 μg/mL) for 5 min. A subsequent PBS wash was executed prior to visualisation, achieved through inverted confocal laser scanning microscopy (LSM 710, Carl Zeiss, Germany). Concurrently, these samples were incubated with primary antibodies targeting IL6, CXCL1, CXCL2, CXCL3, CXCL5, CXCL8, MMP1, MMP3 and MMP12, each at a dilution of 1: 50 (Cloud‐Clone Corp., Wuhan, China). The incubation was conducted overnight at 4°C. Subsequent processing was conducted as per the established immunohistochemistry protocol, culminating in photographic documentation facilitated by an optical microscope (Olympus). To evaluate the occurrence of apoptosis, a TUNEL assay was conducted following the manufacturer's stipulated guidelines (Servicebio, Wuhan, China). Furthermore, normal tissue specimens underwent haematoxylin and eosin (H&E) staining, a standard histological technique employed to assess potential internal lesions.

### 2.9 Transcriptomics analysis

Tumour samples were gathered from both the control and AEF‐treated groups, following a maintained two‐week treatment regimen. Each experimental cohort was composed of three independent triplicates (*n* = 3), ensuring statistical robustness and meaningful insights. Total RNA was extracted from these tumour samples, employing Oligo (dT) magnetic beads to achieve targeted enrichment. The ensuing steps were conducted with a focus on maintaining the quality and efficiency of the processes. Specifically, the Agilent 2100 Bioanalyzer platform was employed to assess both the quantity and labelling efficacy of the synthesised cDNA molecules. Then the synthesised cDNA underwent paired‐end sequencing using the Illumina HiSeq platform. The resulting datasets were subjected to a comprehensive analysis aimed at identifying Differentially Expressed Genes (DEGs). This step facilitates the elucidation of intricate transcriptional variations that characterise the divergence between the control and AEF‐treated groups. The DEseq2 methodology was applied to this endeavour so as to identify genes that show statistically significant expression discrepancies. Simultaneously, a parallel analytical approach was undertaken through the employment of Gene Set Enrichment Analysis (GSEA), which discerns gene sets or pathways that exhibit systematic enrichment with differentially expressed genes.

### 2.10 Quantitative real time‐PCR

Total RNA from tumour tissues was extracted using the Trizol reagent, following the established protocol provided by Invitrogen, USA. Subsequently, 500 ng of total RNA underwent reverse transcription into complementary DNA (cDNA) utilising the qPCR cDNA reverse transcription Mix (Vazyme, China). The cDNA was then subjected to quantitative real‐time polymerase chain reaction (qRT‐PCR) using the ChamQ SYBR qPCR Master Mix (High ROX Premixed) (Vazyme, China). This procedure was carried out in triplicate on the StepOne Real‐time PCR systems (ABI, USA). Non‐template wells were included as negative controls. The calculation of relative mRNA expression levels was accomplished using the 2^−ΔΔCt^ method, facilitating the normalisation of mRNA expression levels to the reference gene glyceraldehyde‐3‐phosphate dehydrogenase.

### 2.11 Statistical analysis

**FIGURE 1:**
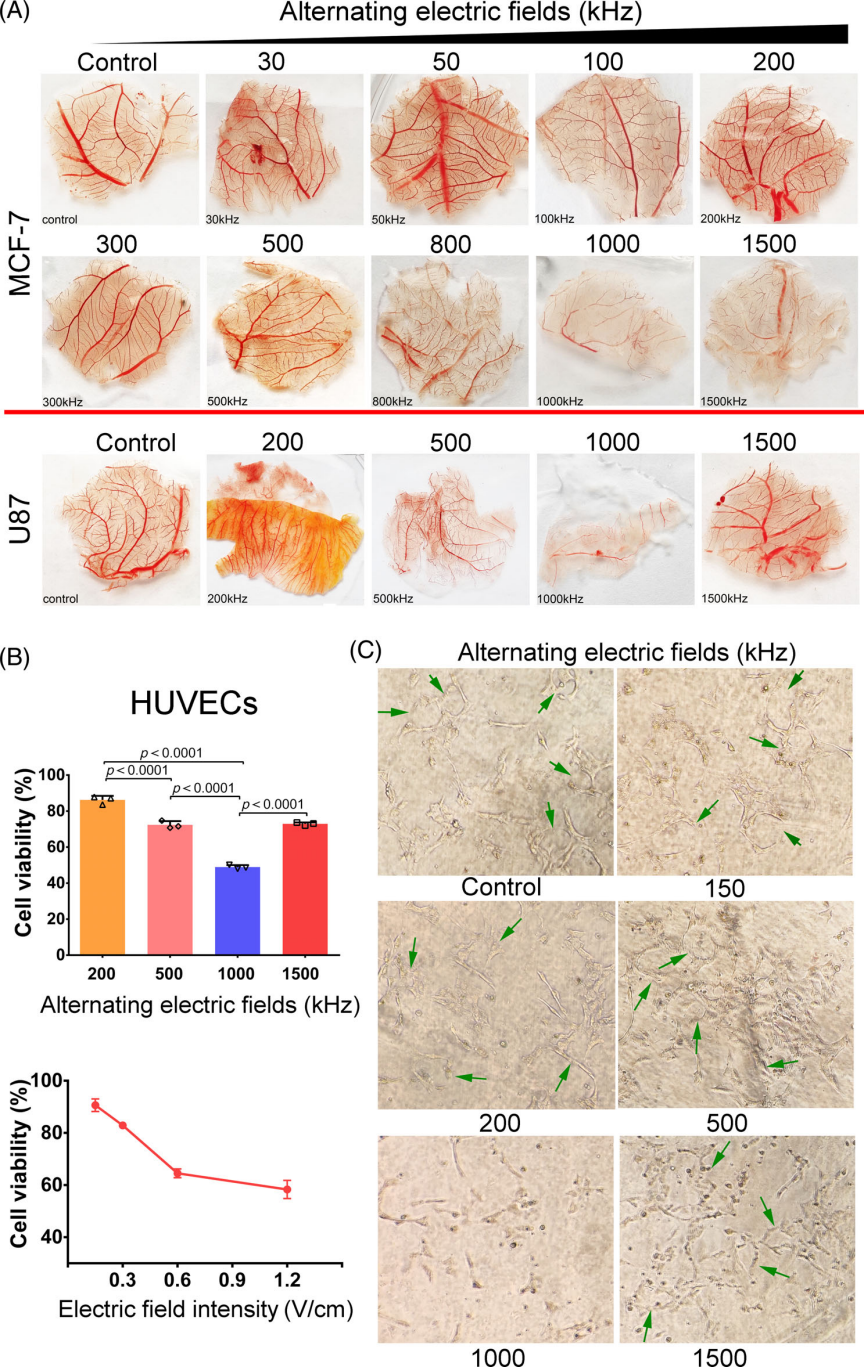
Determining the optimal frequency of alternating electric fields for angiogenesis inhibition. (A) CAM assays were conducted using MCF‐7 and U87MG tumour cells as the stimuli. These cells were subjected to varying frequencies of alternating electric fields for 48 h. The electric field intensity for all treatment groups was maintained at 0.6 V/cm. The control group featured electrodes but did not undergo electric field treatment. The representative image was obtained from three biological assay repeats. (B) Cell viability assay. Left. When the electric fields intensity was set at 0.6 V/cm, 1000 kHz electric fields inhibited the HUVECs to the greatest extent. Right. The growth inhibition effect of 1000 kHz electric fields on the endothelial cells was dose‐dependent. Each symbol represents an average of triplicates. (C) Representative images of HUVEC tube formation assay. To evaluate the impact of alternating electric fields on angiogenesis, HUVECs were cultured for 24 h under the influence of alternating electric fields at frequencies of 200, 500, 1000, and 1500 kHz, all maintained at an electric field intensity of 0.6 V/cm. Subsequently, images of the formed tubes were captured using a microscope. The presented data represent the mean ± SD. *p* values were determined by one‐way ANOVA (B). Data are presented as mean values ± SD. ANOVA, analysis of variance; CAM, Chicken chorioallantoic membrane, HUVEC, human umbilical vein endothelial cells.

Student's *t* test was used for comparison between two groups to determine significant differences (Figure 7A) using SPSS 20.0. Matched groups (three or more) were compared using one‐way Analysis of Variance (ANOVA) in Graphpad Prism 6 (Figures 1B, 3B, and 4B). The presented data were represented as mean ± SD. A significance level of *p* < 0.05 was deemed the threshold for statistical significance.

## 3 RESULTS

### 3.1 1000 kHz alternating electric fields inhibit in vitro angiogenesis

To investigate the optimal parameters of alternating electric fields for anti‐angiogenesis, we used a chicken chorioallantoic membrane (CAM) assay with MCF‐7 breast cancer cells as stimuli. We screened a panel of frequencies to identify the optimal frequency for inhibiting tumour‐induced angiogenesis (Figure S1). Frequencies of 30, 50, 100, 200, 300, 500, 800, 1000 and 1500 kHz were used, with an electric field intensity of 0.6 V/cm for all groups except the control group, which had electrodes but did not receive electric field treatment. After 48 h of treatment, the lower‐frequency groups (30–200 kHz) showed almost intact vessels compared to the control group, while the higher‐frequency groups (300–800 kHz) had fewer capillary branches, with the main trunks remaining less affected (Figure 1A). At 1000 kHz, the main trunks and branches of blood vessels decreased significantly, and the membrane appeared pale (Figure 1A). However, 1500‐kHz AEFs did not produce better results than the 1000‐kHz group. Subsequently, we evaluated the effects of AEFs on angiogenesis in the frequency range of 200 to 1500 kHz in a parallel CAM experiment using U87MG glioblastoma cells as stimuli. Similarly, AEFs at 1000 kHz demonstrated the most potent inhibitory effects on angiogenesis, surpassing other frequencies at the same electric field intensity (Figure 1A). It is noteworthy that the effect of AEFs at 1500 kHz was stronger when MCF‐7 was used as stimulus compared to U87MG. We suppose that it might be due to the different paracrine secretion patterns of these stimuli (Figure 1A). Furthermore, when the electric field intensity exceeded 0.6 V/cm, chicken embryos frequently experienced mortality. Therefore, we determined the optimal parameters for all subsequent experiments to be a frequency of 1000 kHz and an electric field intensity of 0.6 V/cm. We postulated that AEFs' anti‐angiogenic effects were attributed to its cytotoxicity against endothelial cells. Indeed, cell viability tests confirmed that AEFs at 1000 kHz exhibited greater cytotoxicity against human umbilical vein endothelial cells (HUVECs) compared to other frequencies (Figure 1B). Moreover, the cytotoxicity of alternating electric fields at 1000 kHz against HUVECs displayed a proportional increase as the electric field intensity rose. This observation suggests an inhibitory effect of AEFs on vascular endothelial cell growth, consistent with the findings of the CAM assay. (Figure 1B). Then we conducted an endothelial tube formation assay. In contrast to the control group, where endothelial cells formed curved and tubular structures (Figure 1C, green arrows), HUVECs treated with alternating electric fields at frequencies of 200, 500, 1000 and 1500 kHz appeared grey, flat, and exhibited a loss of refraction, and a significant reduction in tube formation; especially, no tube formation was observed in the group treated with 1000 kHz AEFs.

### 3.2 AEFs induces ‘Ball of Yarn’‐like spindle and HUVEC cell death

**FIGURE 2:**
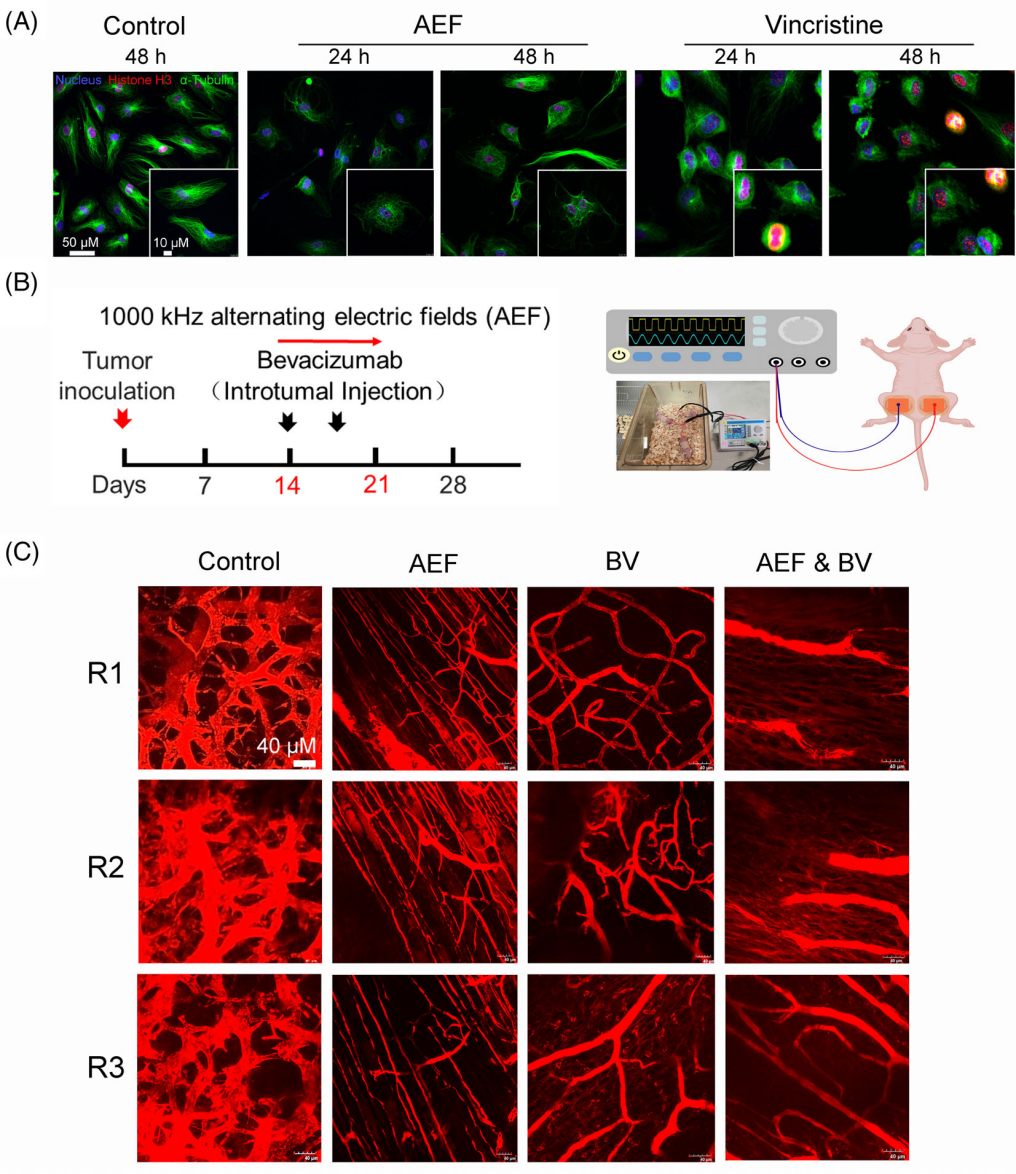
AEFs bring about reduction of tumour vasculature and transformation from a stout interwoven network into an array of slim parallel straight capillaries. (A) Representative images of HUVECs with or without treatment. HUVECs were subjected to 24 or 48 h treatment with AEFs to investigate potential alterations in spindle and chromosome morphology. As a positive control, 5 μM of Vincristine was employed. The cellular spindle and chromosome structures were visualised using specific markers: Anti‐α‐Tubulin (green) for spindle identification and Anti‐Histone H3 (phospho S10) (red) for chromosome labelling. (B) Experimental system overview. A schematic diagram depicting the experimental setup is presented. Mice were subjected to AEFs treatment, followed by i.t. administration of Bevacizumab (administered twice) or a combination of both treatments. This regimen was conducted over a 7‐day period once the tumours reached a size of 100–200 mm^3^. Each experimental group contains three mice. (C) Representative images of tumour vasculatures with or without treatment. Representative longitudinal images were captured for the control, AEFs‐treated, Bevacizumab‐treated, and AEFs & Bevacizumab‐treated groups (*n* = 3 animals per group). At the culmination of the experiment, mice were i.v. injected with 100 μL of Texas Red labelled dextran (10 mg/mL) to label tumour vasculature. Subsequently, tumour blood vessels were observed and documented utilising a Two‐photon Microscope. The notations R1, R2 and R3 correspond to distinct individual mice. These results are representative of three independent experiments with similar results. BV, Bevacizumab; AEFs, 1000 kHz alternating electric fields. AEF, alternating electric fields; ANOVA, analysis of variance; HUVEC, human umbilical vein endothelial cells.

As the mechanism of TTFields involves spindle formation and mitotic destruction,^19^ we sought to determine whether AEFs induced HUVEC cell death by affecting spindle formation. Our results revealed that 48‐hour treatment with AEFs induced a distinct ‘ball of yarn’‐like spindle structure, contrasting with the shuttle‐shaped spindles observed in the control group (Figure 2A). The spindle structure induced by AEFs resembled the spindles in the vincristine‐treated group, suggesting a possibly common mode of action (Figure 2A). Vincristine is known to bind to microtubule proteins, impeding the polymerisation of microtubule dimers, and thereby preventing chromosome separation during metaphase.^20^ These findings suggest that AEFs exerted their inhibitory effects on HUVEC growth by disrupting spindle formation. We did not observe abnormal behaviour of histone H3‐labelled chromosomes among the experimental groups (Figure 2A). Furthermore, immunocytochemistry demonstrated that AEFs induced HUVEC cell death (Figure S2), which aligned with the results from the cell viability assay (Figure 1B). These results imply that AEFs induces ‘Ball of yarn’‐like spindle formation, disrupts HUVEC cell division and ultimately leads to cell death.

### 3.3 AEFs reduce tumour vasculature and transform it from a stout, interwoven network into an array of slender, parallel, straight capillaries

While TTFields have shown remarkable success in glioblastoma treatment, there are very few reports on their in vivo anti‐angiogenesis effects. In contrast, in our study, malignant tumour cells only served as stimuli, while the proliferating endothelial cells and the vasculature were the research objects. Therefore, once the ideal frequency of AEFs was determined, we concentrated our efforts on studying endothelial cells and angiogenesis, mainly utilising U87MG as the stimuli for the subsequent in vivo experiments, unless otherwise specified. Building upon the promising results from CAM and tube formation assays, we established a U87MG glioblastoma mouse model to evaluate the in vivo anti‐angiogenesis effect of 1000 kHz AEFs. The tumour‐bearing mice were intratumorally (*i.t*) injected with PBS or Bevacizumab, and then subjected to AEFs treatment by adhering insulated copper plate electrodes to the tumour surface (Figures 2B and S1). After 1 week of treatment, ghost imaging was employed using Texas Red labelled Dextran to examine the morphological characteristics of tumour blood vessels. Representative images of the tumour vasculature from the control, AEFs, Bevacizumab and AEFs & Bevacizumab groups were analysed. The blood vessels in the control groups displayed a stout, interwoven network shape, with many haphazard interconnections (Figure 2C). In the Bevacizumab group, the vasculature shape resembles that of the control, but the diameters of the vessels shrunk obviously and the capillaries became fewer. However, in the AEFs group, the tumour vessels turned into an array of slender, parallel, straight capillaries.^21^ Interestingly, the parallel capillaries were strictly perpendicular to the electric field direction. In addition, we noted that many capillaries were disrupted into dozens of fragments (Figure 2C). In the AEFs & Bevacizumab group, the tumour vasculatures showed the characteristics of both Bevacizumab and AEFs groups, that is both shrunk vessel trunks and parallel, straight capillaries were present (Figure 2C).

**FIGURE 3:**
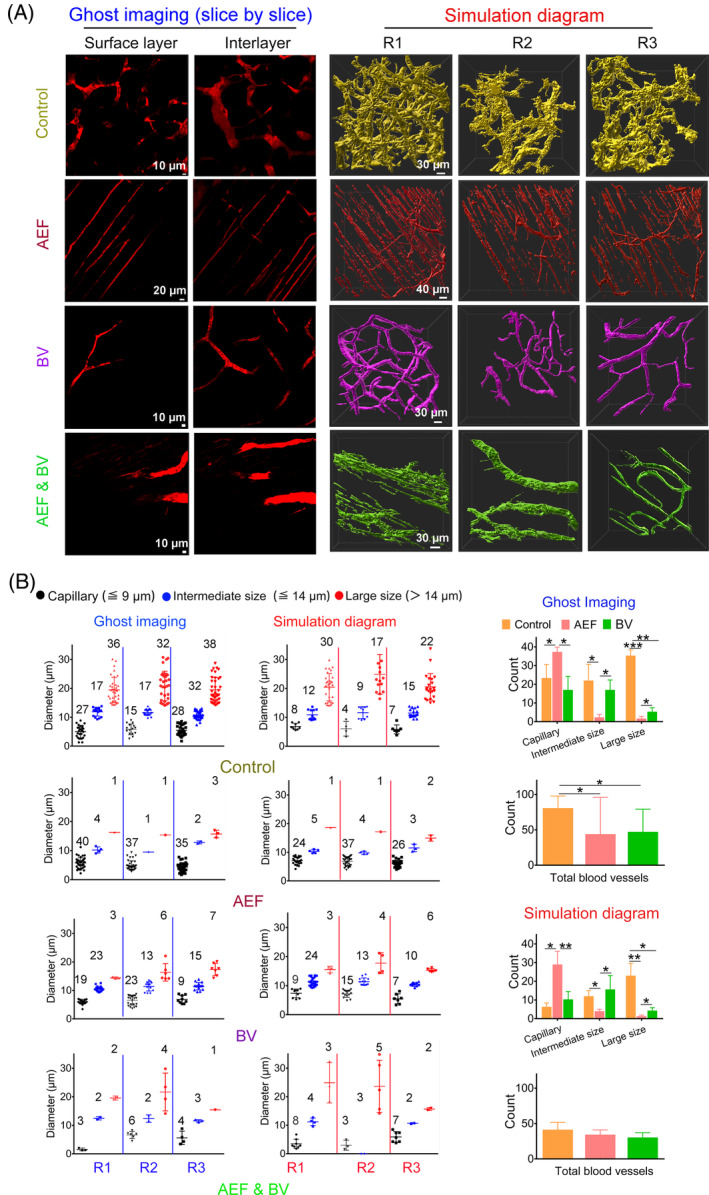
Quantification of vessel numbers and diameters by size and type. (A) Representative images of tumour vessels, acquired in a slice‐by‐slice manner using a Two‐photon Microscope and the projection of 3D images generated using ImarisViewer 9.8 software. The number and diameter of these vessels within the control, AEFs‐treated, Bevacizumab‐treated, and AEFs & Bevacizumab‐treated groups were independently assessed using a statistical approach. Data were derived from three mice per experimental group. (B) Quantitative analysis of vessel types. The total counts of capillary‐sized vessels (≦9 μm) (black dots), intermediate‐sized vessels (≦14 μm) (blue dots) and large‐sized vessels (>14 μm) (red dots) were quantified across different experimental groups and subjected to comparative analysis. The Arabic numerals on the dot groups indicate the number of vessels. R1, R2 and R3 stand for three biological repeats. The horizontal bars represent group averages. BV, Bevacizumab; AEFs, 1000 kHz alternating electric fields. Data are presented as mean values ± SD. Statistical differences of p values were determined by One‐way ANOVA (B). AEF, alternating electric fields; ANOVA, analysis of variance; HUVEC, human umbilical vein endothelial cells.

To further analyse and quantify these tumour vasculatures in detail, 3D vessel analysis was conducted to produce simulation diagrams for each ghost image, offering a panoramic view of tumour blood vessels (Figure 3A). Subsequently, we conducted quantitative analysis of tumour blood vessels using both ghost imaging (slice by slice) and simulation diagrams. Ghost imaging allowed us to assess the number and diameter of blood vessels in each slice of the tumour samples. The vessels were classified into three types based on their diameter: ‘capillary’ (<9 μm), ‘intermediate size’ (9–14 μm) and ‘large size’ (>14 μm). In Figure 3A, representative images from the surface layer and interlayer in tumours were displayed for all groups. Each ghost imaging or simulation diagram contained data from three independent mice (R1, R2 and R3), and the vessel numbers for each type were listed above the chart points (Figure 3B). We observed that the number of vessels in the simulation diagrams was consistent with the ghost imaging results for all treatment groups, but not the control group (Figure 3B). The leaky characteristic of tumour vasculature in the control group resulted in the release of the imaging agent into the surrounding microenvironment, leading to a noise background that made it challenging for the analysis software to distinguish vessels from the background, especially capillaries (Figure 2C). To further compare the mean numbers of the three vessel types among groups, we analysed the data from ghost imaging. AEFs led to a substantial increase in capillaries (37.33 ± 2.52), significantly more than the control (23.33 ± 7.23, *p =* 0.0167) and Bevacizumab groups (17.0 ± 7.21, *p =* 0.015) (Figure 3B). On the other hand, the AEFs group exhibited remarkably fewer intermediate size vessels (2.3 ± 1.5) than the control (22.0 ± 8.66, *p =* 0.0144) and Bevacizumab (17.0 ± 5.29, *p =* 0.0449) groups (Figure 3B). It's worth noting that the AEFs group had almost no large vessels. Furthermore, the total vessel counts in the control group exhibited a significant difference when compared to the AEFs and Bevacizumab groups as demonstrated in ghost imaging (Figure 3B). No significant difference was observed in total vessel counts between the AEFs and Bevacizumab groups. The comprehensive analysis of ghost imaging and simulation diagrams elucidated the effects of AEFs on tumour blood vessels, demonstrating a reduction in vessel number, decreased vessel diameter and transformation of the tumour vasculature shape. Notably, AEFs treatment achieved similar anti‐angiogenic efficacy as that of *i.t*. injected Bevacizumab (Figures 2C and 3), highlighting its promising potential as an effective anti‐angiogenesis therapy.

### 3.4 AEFs inhibit angiogenesis and tumour growth and synergises with bevacizumab

**FIGURE 4:**
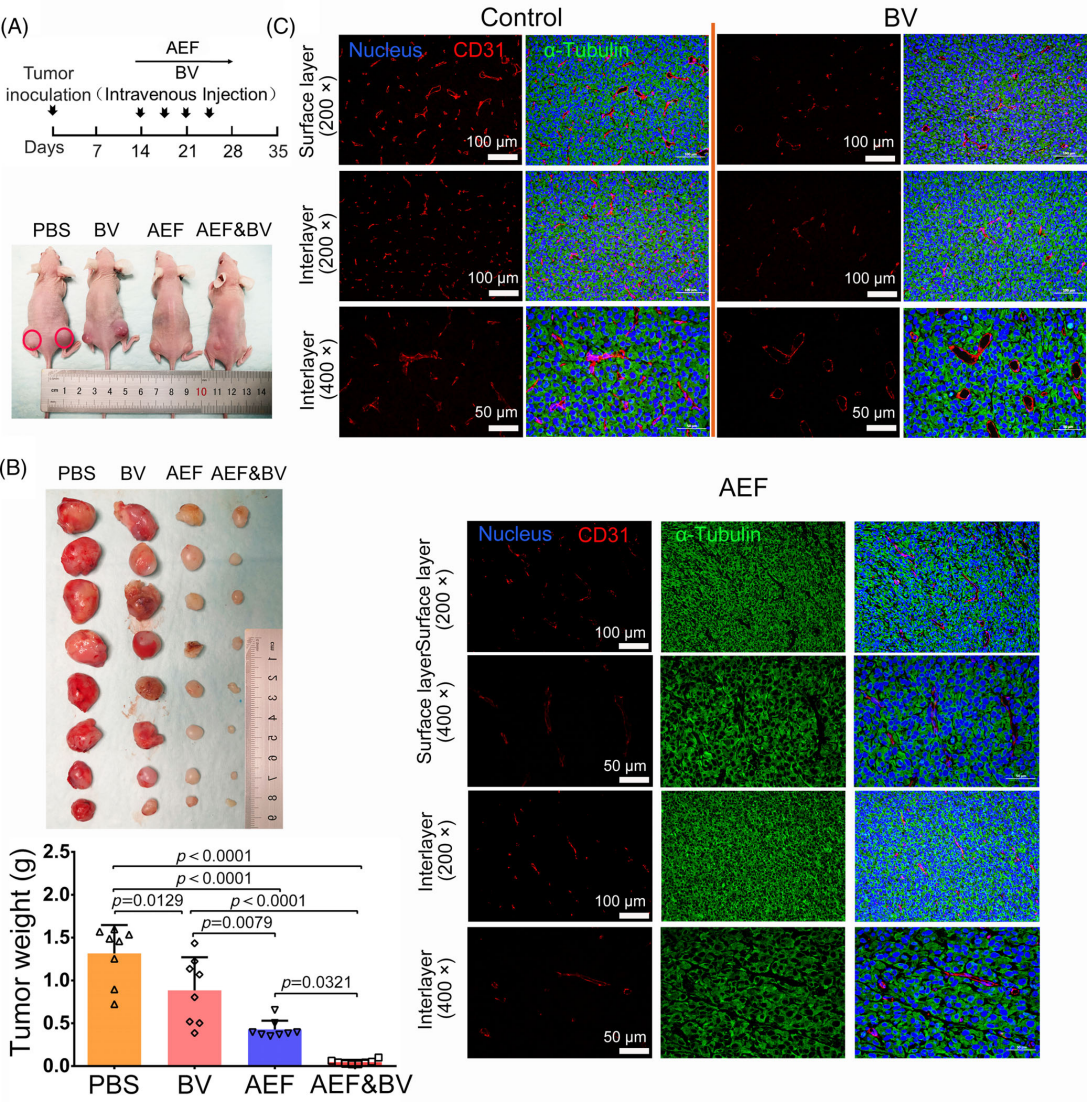
AEFs inhibits angiogenesis and tumour growth and synergises with bevacizumab. (A) Experimental design overview and representative images of mice with U87MG tumours at the end of treatment. A schematic illustration outlines the experimental setup. Mice bearing U87MG tumours were subjected to treatment involving 1000 KHz alternating electric fields (AEFs), i.v. administration of Bevacizumab or PBS or a combined therapeutic approach (AEFs & BV) spanning a duration of 14 days. (B) Tumour weight analysis. Following the treatment regimen, the tumours were harvested, and their respective weights were recorded. The weight of the obtained tumours was assessed as an indicator of treatment effectiveness. Each mouse bears two tumours. There are a total of four nude mice in each group (PBS, BV, AEFs, and AEFs & BV). The presented data represent the mean ± SD. (C) Representative images of tumour vasculature with or without treatment. Fluorescent immunohistochemistry was employed to visualise the tumour microenvironment in the coronal plane of tumour tissues that is parallel to the copper plate electrodes. This imaging technique allowed for the visualisation of 4′,6‐diamidino‐2‐phenylindole (DAPI)‐stained nucleus (blue colour), CD31‐labelled vessels (depicted in red), and α‐Tubulin‐marked spindles (represented in green). These results were obtained from three independent experiments. BV, Bevacizumab; AEFs, 1000 kHz alternating electric fields. *P* values were obtained using one ANOVA (B). ANOVA, analysis of variance. PBS, Phosphate Buffered Saline, DAPI, 4',6‐Diamidino‐2‐phenylindole.

The efficacy of AEFs in inhibiting the growth of tumour was further evaluated by extending the treatment period and using intravenous (*i.v*.) injected bevacizumab as a positive control, considering the standard drug administration in clinical cancer therapy (Figure 4A). After 14 days of treatment, the tumour volumes of AEFs group (0.42 ± 0.1 g, *p <* 0.0001), bevacizumab group (0.88 ± 0.39 g, *p =* 0.0129), and AEFs & Bevacizumab group (0.045 ± 0.026 g, *p <* 0.0001) were significantly smaller than the control (1.31 ± 0.33 g) (Figure 4A). It's worth noting that the tumour volume of AEFs & Bevacizumab group is much smaller than AEFs (*p =* 0.0321) or bevacizumab (*p =* 0.0079) alone, implying that AEFs synergise with bevacizumab in term of anti‐angiogenesis and tumour suppression, taking into account of the above‐mentioned vessel analysis data. We noted that the left and right tumours are not identical for each mouse, despite efforts to maintain uniform conditions during tumour induction. This phenomenon may result from the tumour heterogeneity and anatomical asymmetry such as local tissue microenvironments and subtle differences in blood flow.

**FIGURE 5:**
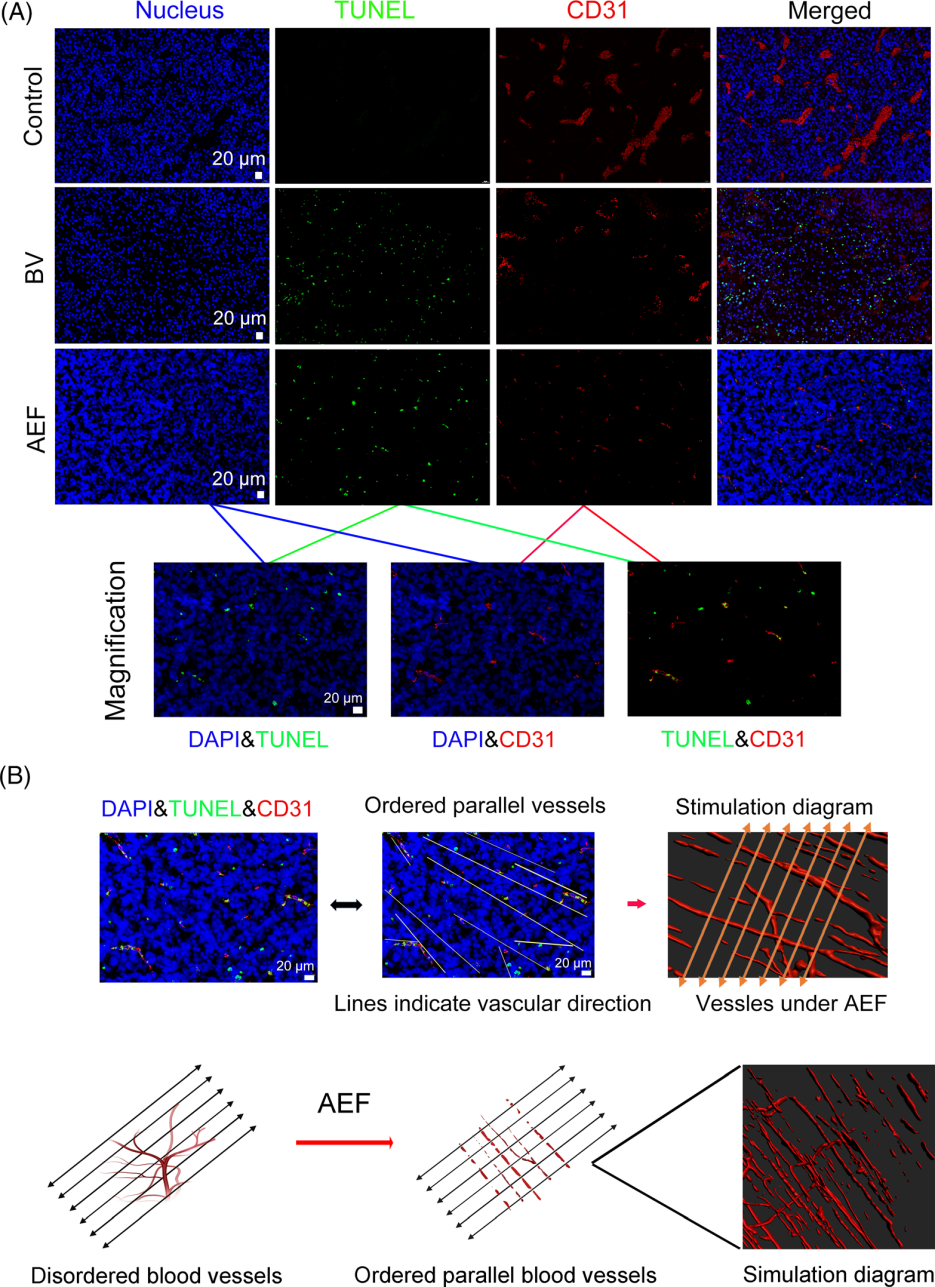
AEFs induce endothelial cell apoptosis and transformation of tumour vasculature into parallel capillaries. (A) Detection of apoptotic cells. Simultaneously, a TUNEL assay was conducted to identify apoptotic cells within the tumour tissues. Apoptotic cells are visualised in green, while the vascular structures are shown in red. (B) Overlapping apoptotic cells and blood vessels. Apoptotic cells were found to overlap with the blood vessels within tumours that are oriented perpendicular to the direction of the electric field. These images are indicative of outcomes obtained from three independent experimental trials. DAPI, 4′,6‐Diamidino‐2‐phenylindole; BV, Bevacizumab; AEFs, 1000 kHz alternating electric fields. AEF, alternating electric fields.

To further examine the distribution and organisation of the ‘parallel capillary’ pattern caused by 1000 kHz AEFs, we conducted immunohistochemistry on the tumour samples. Given that the electric field direction was known to be perpendicular to the tumour's coronal planes (Figure 2C), we obtained paraffin sections in both coronal and transverse orientations for immunohistochemistry. This allowed us to closely examine the arrangement of blood vessels in different planes and gain a comprehensive understanding of the impact of AEFs on the tumour vascular network. In the control group, tumour vasculatures in the interlayer and surface layer appeared large, expanded, and randomly dispersed, without any unified direction in both coronal and transverse orientations (Figures 4C and S3). The vessel morphology and distribution in the bevacizumab group were similar to the control in both coronal and transverse orientations, except that they were fewer and shrunk (Figures 4C and S3). In contrast, the blood vessels of the AEF‐treated tumour exhibited a uniform direction with discontinuous punctuate structure in coronal orientation (Figure 4C), which aligns with the ghost imaging results (Figure 2C). Nevertheless, they displayed a random dispersion pattern in the transverse orientation of the tumour (Figure S3). Moreover, the number of blood vessels in the surface and interlayer was reduced in the AEFs group compared to the control group (Figure 4C). The tumour cells stained with DAPI (nucleus) and α‐tubulin did not show changes among the three groups, indicating that AEFs primarily affected the vascular endothelial cells rather than tumour cells (Figure 4C). This observation was further confirmed by TUNEL assay, which showed that bevacizumab induced apoptosis in both tumour cells and vascular endothelial cells, while in the AEFs group, most apoptotic cells (green) overlapped with the CD31‐labelled vascular endothelial cells (Red) (Figure 5A), implying that 1000 kHz AEFs preferentially induce apoptosis of vascular endothelial cells rather than the tumour cells. By applying trendlines to interconnect the punctuated vessels, we generated a simulation diagram resembling ghost imaging, in which the capillaries within tumours are oriented perpendicular to the direction of the electric field (Figure 5B). In summary, the ghost imaging, immunohistochemistry results, and TUNEL assay provide insights into the effects of AEFs on tumour blood vessels.

To ensure that our findings were not influenced by potential variations in different tumour cell sorts as stimuli, we then standardised our in vivo studies using MCF‐7 xenograft tumours to evaluate the anti‐angiogenic effects of AEFs. Two‐photon microscopy revealed that the MCF‐7 tumour vasculature initially exhibited a dense, interwoven structure. AEF treatment induced a marked transformation, resulting in an array of slender, parallel, straight capillaries oriented perpendicular to the electric field (Figure S4A). The tumour size of the AEF group is much smaller than the control (Figure S4B). Immunohistochemistry staining demonstrated a pronounced abundance of CD31+ vessels in MCF‐7 xenograft tumours, while these vessels were markedly reduced in AEF‐treated tumours (Figure S4C). A TUNEL assay was conducted to identify apoptotic cells within the tumour tissues. Apoptotic cells in the AEF group were found to overlap with the blood vessels that are oriented perpendicular to the direction of the electric field (Figure S4D).

Meanwhile, we found that AEFs treatment yielded no discernible indications of organ impairment (heart, liver, spleen, kidney and lung) (Figure S5).

### 3.5 Downregulation of chemokines and matrix metalloproteinase contribute to the anti‐angiogenesis effect of AEFs

**FIGURE 6:**
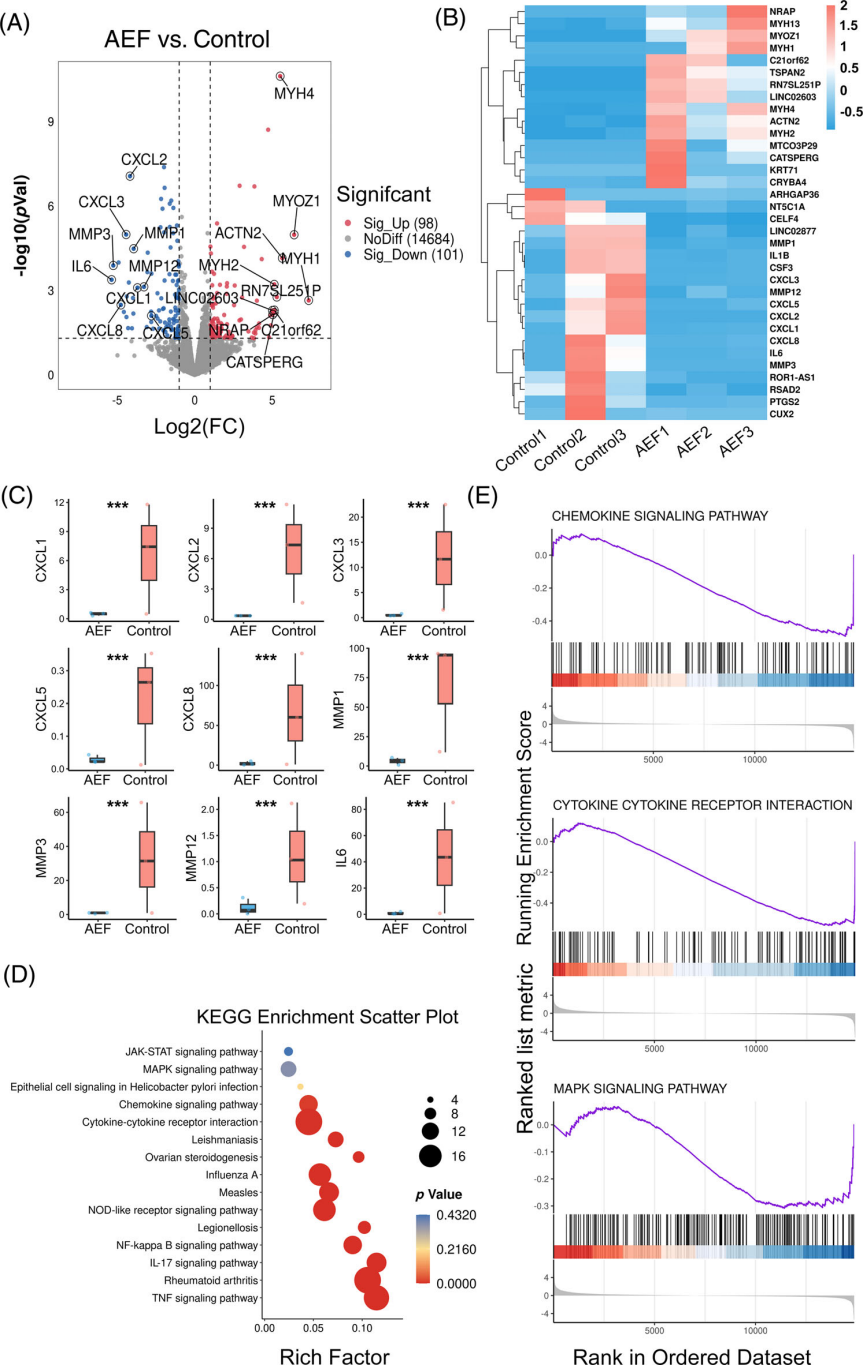
AEFs downregulate angiogenesis‐related chemokines and matrix metalloproteinases. **A** A volcano plot shows the results of transcriptomic analysis comparing the control and AEFs‐treated groups, as previously described in Figure 4A (*n* = 3 biological replicates each). Green dots and grey dots represent the downregulation genes and no different genes, respectively, while red dots stand for the upregulation genes after AEFs treatment in comparison with the control group. (B) Differentially expressed genes between the control and AEFs‐treated groups are depicted in a hierarchical clustering heatmap, highlighting patterns of gene expression alteration. Red represents high gene expression, while blue represents low gene expression (*n* = 3 biological replicates each). (C) Relative abundance of differentially expressed genes associated with angiogenesis (*n* = 3). Box‐and‐whiskers plots represent the interquartile ranges (25th through 75th percentiles, boxes), medians (50th percentiles, bars within the boxes), the 5th and 95th percentiles (whiskers below and above the boxes). (D) A scatter plot demonstrates the results of KEGG pathway enrichment analysis, derived from the transcriptomic analysis of the control and AEFs‐treated groups as detailed in (A) (*n* = 3). The colour depth and bubble size indicate –ln (p) values and impact of the pathway. (E) Utilizing Gene Set Enrichment Analysis (GSEA), the control and AEFs‐treated tumours from (A) were subjected to gene set enrichment analysis, revealing significant enrichment patterns (*n* = 3). These outcomes are representative of the similar findings from three independent trials. Raw data of transcriptome can be acquired from China National Center for Bioinformation (ID: PRJCA019083; https://www.cncb.ac.cn). AEF, alternating electric fields. KEGG, Kyoto Encyclopedia of Genes and Genomes.

Apart from the discernible impact of AEFs in hindering the mitotic division of endothelial cells, we propose that AEFs might affect the microenvironmental molecules involved in tumour angiogenesis. Therefore, we performed a comprehensive bioinformatics analysis of the transcriptomic data from tumours in the control and AEFs‐treated groups (Figure 4A). The volcano plot displayed the differential gene expression between the two groups, revealing that AEFs treatment upregulated 98 genes and downregulated 101 genes compared to the control (Figure 6A). Hierarchical clustering heatmap analysis further highlighted the main differential genes (Figure 6B). Among the upregulated genes in the AEFs group were myosin heavy chain (MYH)1, MYH2, MYH4, MYH13, NRAP (Nebulin‐related actin‐binding protein), LINC02603, tetraspanin 2 (TSPAN2) and actinin alpha 2 (ACTN2), which are primarily associated with muscle function and cell movement.^22^, ^23^ The re‐expression or upregulation of these genes has been linked to muscle regeneration or damage, similar to the effects observed with chronic electrical stimulation.^22^ This may partly explain the phenomenon of parallel capillary formation in the AEF‐treated tumours, which may involve cell movement in a given direction.^21^ Moreover, AEFs treatment led to significant downregulation of chemokines (CXCL1, CXCL2, CXCL3, CXCL5 and CXCL8) and matrix metalloproteinases (MMP1, MMP3 and MMP12) compared to the control (Figure 6C). Notably, members of the CXC chemokine family, including CXCL1, CXCL2, CXCL3, CXCL5 and CXCL8, function through the activation of CXCR1 and CXCR2, playing a key role in promoting tumour angiogenesis.^24^ Similarly, matrix metalloproteinases MMP1, MMP3 and MMP12 are crucial regulators of tumour angiogenesis, vascular structure, permeability and integrity.^25^ The downregulation of these chemokines and matrix metalloproteinases contributed to the anti‐angiogenic effects of AEFs. Additionally, AEF treatment reduced interleukin 6 (IL‐6) levels, a characteristic feature of the tumour microenvironment that promotes tumorigenesis by regulating various cancer hallmarks and multiple signalling pathways, including proliferation, apoptosis, angiogenesis and metastasis.^26^ The decrease in IL‐6 levels indicated a downregulation of cancer‐associated characteristics and restrained tumour growth. However, other important angiogenesis‐related factors, such as VEGF and HIF‐1α, showed no significant differences between the AEFs and control groups. This suggests that the anti‐angiogenesis effect of 1000 kHz AEFs is VEGF‐independent, making it a complementary therapy to bevacizumab. Furthermore, Kyoto Encyclopedia of Genes and Genomes pathway analysis revealed significant alterations in pathways known for their anti‐tumour and anti‐angiogenesis capabilities, including MAPK, cytokine‐cytokine receptor interaction, NOD‐like receptor, chemokine signalling and TNF signalling pathways (Figure 6D). Gene Set Enrichment Analysis (GSEA) of tumour gene data demonstrated downregulation of MAPK signalling pathway, cytokine‐cytokine receptor interaction, chemokine signalling pathway and JAK STAT signalling pathway in the AEFs group compared to the control (Figures 6E and S6). The downregulation of these signalling pathways supports the inhibitory effects of AEFs on tumour angiogenesis.

**FIGURE 7:**
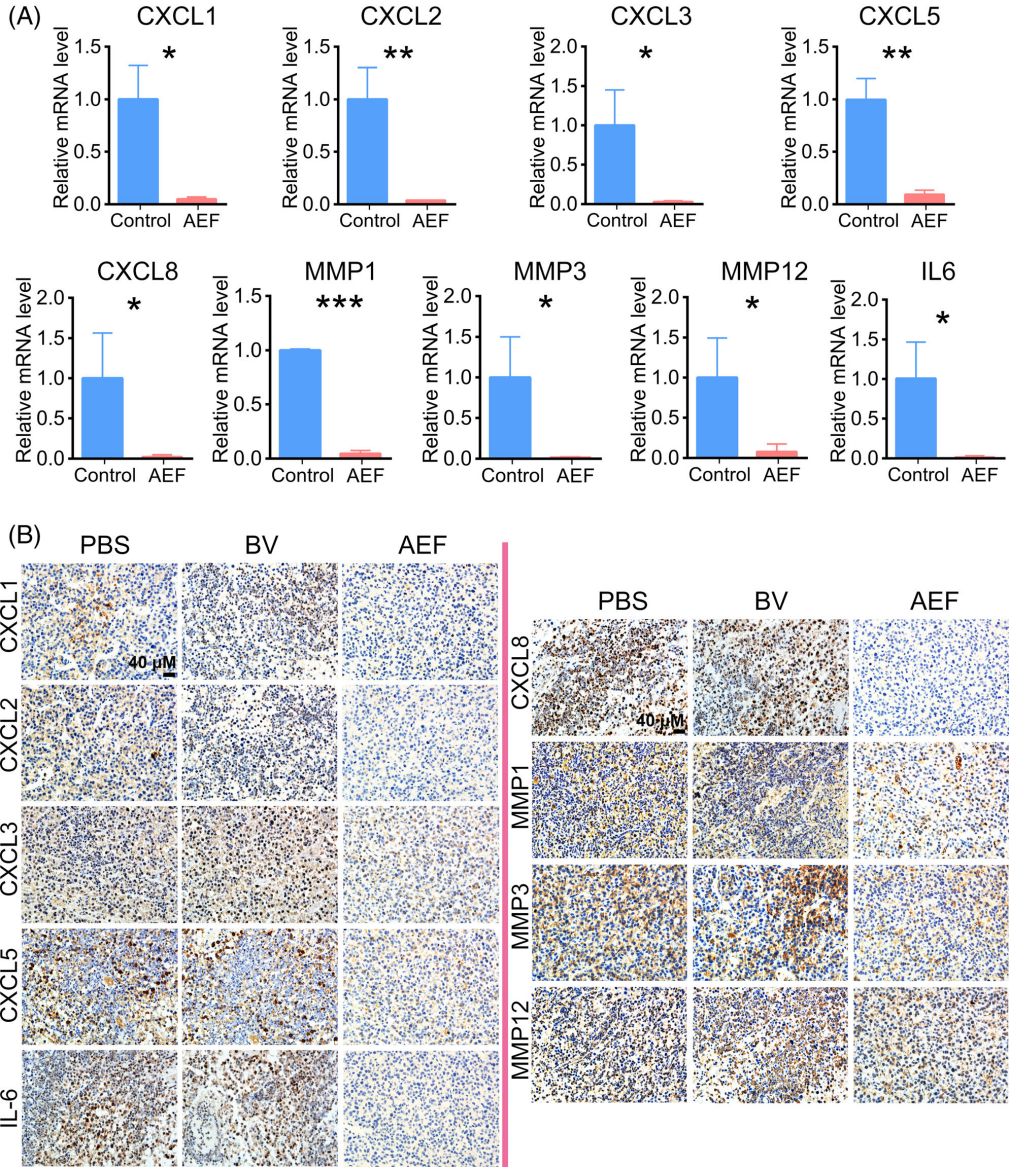
Validation of gene expression pattern through Quantitative Real‐time polymerase chain reaction (qPCR) and immunohistochemistry. (A) qPCR analysis. Total mRNA was isolated from the tumours as described in Figure 4A. Subsequently, qPCR was performed, with data analysed by normalisation to GAPDH expression levels. Data are shown as the mean ± SD values (*n* = 3 biological replicates each). (B) Immunohistochemistry Evaluation. Tumour samples outlined in Figure 4A underwent immunohistochemistry analysis. The pictures represent immunohistochemistry results derived from an average of three biological replicates. *p* values were obtained using Student's *t* test (A). AEF, alternating electric fields; BV, Bevacizumab. GAPDH, glyceraldehyde‐3‐phosphate dehydrogenase.

The major gene expression changes in AEF‐treated tumours were further confirmed by real‐time PCR using their specific primers (Table S1) and immunohistochemistry. The results showed that many key angiogenesis‐related genes were downregulated in the AEFs group compared to the control (Figure 7A). In particular, the mRNA levels of CXCL1 (*p =* 0.0112), CXCL5 (*p =* 0.0039), and MMP12 (*p =* 0.041) in the control group were over ten times higher than those in the AEFs group, while CXCL2 (*p =* 0.0091), CXCL3 (*p =* 0.0267), CXCL8 (*p =* 0.0463) and MMP1 (*p =* 0.0002) showed over twenty times higher expression in the control group. Furthermore, the mRNA levels of IL‐6 (*p =* 0.0266) and MMP3 (*p =* 0.0336) in the AEFs group were remarkably lower than those in the control group, exhibiting 61 and 71 times reduction, respectively. Immunohistochemistry further validated the downregulation of proteins for these genes (Figure 7B). CXCL1, CXCL2 and CXCL8 proteins were almost completely removed from the tumours after AEFs treatment. Although some residual proteins of CXCL3, CXCL5, MMP1, MMP3 and MMP12 were still detected, their levels were significantly decreased due to AEFs treatment. Overall, these findings provide evidence that AEFs reduced the expression of angiogenesis‐related chemokines and matrix metalloproteinase in glioblastoma tumours, thereby downregulating the signalling pathways and leading to the inhibition of tumour angiogenesis.

## 4 DISCUSSION

Emerging evidence demonstrates that tumour vasculature not only promotes tumour progression and metastasis but also decreases the efficacy of anticancer drugs over time.^27^ It's reported that bevacizumab enhances cancer chemotherapy by inhibiting angiogenesis and normalising tumour blood vessels to bolster drug penetration.^14^ Recent clinical studies have witnessed the utilisation of diverse anti‐angiogenic drugs, including monoclonal antibodies, small‐molecule multi‐target tyrosine kinase inhibitors, such as sorafenib, sunitinib, regofinil, arotinib, and apatinib and endostatin.^28^ However, these agents inevitably produce side effects on normal cells. In contrast, TTFields, functioning as a regional physical therapy, demonstrate remarkable efficacy and safety in treating gliomas in clinical realms.^29^, ^30^ Inspired by the action of TTFields on proliferating cancer cells, this study found that alternating electric fields at 1000 kHz specifically inhibit the proliferating vascular endothelial cells. Because vascular endothelial cells in adult normal tissues are generally quiescent, except for a few organs such as the uterus,^31^ and the effect of electric fields is strictly limited to the area between the pair of plate electrodes, with extremely low electric field intensity outside this area, this novel anti‐angiogenesis method should be able to preserve the physiological integrity of normal organs.

Successful clinical applications of TTFields have been observed in glioblastoma and malignant pleural mesothelioma patients, with only mild adverse side effects,^30^, ^32^ augmenting the efficacy of chemotherapy,^33^, ^34^ immunotherapy,^10^ and radiotherapy.^35^ It is noteworthy that the optimal anti‐proliferative frequencies of TTFields are varying across different cancer cell lines.^1^ For instance, the efficacy of 200 kHz has been established for human glioblastoma (U87MG, LN229, A172, LN‐18, LN‐428, LN‐319, T98G and LN‐308), human ependymoma DKFZ‐EPN1, pancreatic cancer cell lines (CFPAC‐I and HPAF‐II), lewis lung carcinoma (LLC‐1) and human fibroblast; while 150 kHz is effective for human osteosarcoma (U2OS, KHOS/NP), human lung squamous cell carcinoma (H520), human hepatocellular carcinoma (HEPG2) and murine mammary carcinoma cells (4 T1).^1^ However, there are very few reports concerning about the optimal anti‐angiogenic frequency of alternating electric fields that should target the proliferating vascular endothelial cells rather than tumour cells. Our study revealed that alternating electric fields at 1000 kHz preferentially exert an anti‐angiogenic effect. Given the different frequencies of angiogenesis‐targeted AEFs and TTFields, we propose a new strategy: employing alternating electric fields at both 1000 kHz and 200 kHz, with a rotation period of 1–5 s, to concurrently inhibit tumour growth and angiogenesis. We hypothesise that combining 1000 and 200 kHz electric fields may have better therapeutic efficacy than 200 kHz TTFields alone in future clinical trials for glioblastoma and other malignant tumours.

It's reported that TTFields at 100 kHz modulate the cellular morphology of murine cerebellum‐derived vascular endothelial cells, resulted in a temporary disruption of the blood–brain barrier, thereby enhancing the permeability and efficacy of paclitaxel in the treatment of glioblastoma.^9^, ^36^ In addition, several previous investigations have unveiled that TTFields suppress vascular tube formation and inhibit angiogenesis by downregulating angiogenesis‐associated genes, such as MMP9, MMP2, VEGF and HIF1‐α.^11^, ^12^ Our findings differ from previous reports in that 1000 kHz AEFs specifically induced vascular endothelial cell apoptosis, not only reducing tumour vessel density but also transforming the vasculature from a stout, interwoven network into an array of slim, parallel, straight capillaries that are strictly perpendicular to the direction of the electric field. To our best knowledge, this is the first time that tumour vasculature was arranged in a given direction under external forces. In addition, we found that the anti‐angiogenesis effect of 1000 kHz AEFs is independent of VEGF; therefore, it is just complementary to bevacizumab based on their mechanisms. After AEFs treatment, we observed downregulation of IL‐6, a versatile cytokine with both pro‐inflammatory and anti‐inflammatory functions. IL‐6 plays a pivotal role in orchestrating the sprouting angiogenic response of endothelial cells, and its depletion impairs the formation of tube‐like structures.^37^ Chemokines are potent chemoattractants that play a vital role in directing the migration of diverse cell types, including endothelial cells essential for angiogenesis.^24^ Our study revealed that AEFs treatment inhibits the expression of chemokines, thereby impeding the recruitment and mobilisation of endothelial cells to the tumour site. Furthermore, AEFs treatment reduced the expression of MMPs, an enzyme family that plays a pivotal role in degrading the extracellular matrix, a crucial step in the establishment and remodelling of blood vessels during angiogenesis.^25^ By inhibiting MMP expression, AEFs hinder the ability of endothelial cells to invade and remodel the surrounding tissue, further inhibiting the angiogenic process.

Preceding research has underscored the significance of the spatial arrangement of TTFields transducer arrays in impeding the migration of cancer cells.^38^ Optimal reduction in cancer cell migration velocity has been observed when these arrays are positioned perpendicular to the leading edge of cell movement. In our study, we observed AEFs transformed the interwoven network of tumour vessels into parallel straight capillaries perpendicular to the direction of the electric field. (Figure 2C). In addition, we revealed substantial overlap between apoptotic cells and CD31‐labelled blood vessels within AEFs‐treated tumours (Figure 5). This finding demonstrates that vascular endothelial cells that oriented perpendicular to the electric field direction are less susceptible to the effects of AEFs. We suppose that two pairs of mutually perpendicular electrodes might further destroy the tumour vasculature, as the electric field direction will change continuously. In clinical practice, TTFields are applied to glioblastoma by fixing two pairs of electrodes around the brain at a 90° vertical orientation. In this study, we affixed only one pair of insulated copper plate electrodes to the tumour surface to conduct AEFs therapy, which was limited by the small size and irregular shape of the animal tumour. For technical reasons, currently we are unable to attach two pairs of plate electrodes perpendicular to each other around the mouse xenograft tumour. In future studies, we will seek to develop large animal models that can be used for a comprehensive evaluation of the anti‐angiogenesis effect of 1000 kHz AEFs.

In addition, our findings indicate that the target of 1000 kHz AEFs is vascular endothelial cells and angiogenesis. Besides in malignant tumours, active angiogenesis is also involved in some benign lesions, such as arteriovenous malformation, hemangioblastoma and hemangiopericytoma. Therefore, we further propose that 1000 kHz AEFs might be extended to the research of those benign vascular lesions; and this is also a major difference from TTFields. Although our study shows promising results for the anti‐angiogenic effects of AEFs, our findings are based on in vitro experiments and animal tumour models. Further clinical trials are needed to validate the efficacy of AEFs in real‐world settings. Furthermore, we endeavoured to establish an animal tumour model using patient‐derived primary glioma cells, but were not successful. This aspect will be addressed in future attempts.

## 5 CONCLUSIONS

Our study reveals that AEFs treatment is a promising therapeutic avenue for impeding tumour growth by inhibiting angiogenesis, transforming tumour vasculature, and inducing apoptosis of vascular endothelial cells. This distinct anti‐angiogenic effect of AEFs makes it as a valuable tool in the comprehensive management of malignant tumours.

## AUTHOR CONTRIBUTIONS

XSZ and YLZ designed the study. LS, SL, YLW and YY performed all experiments except for ghost imaging and transcription analysis. YTL performed ghost imaging and data analysis. YLZ conducted immunochemistry. YLZ performed transcriptomics analysis. XSZ and YLZ provided funding. YLZ, LS, YLW and YY wrote the initial draft, with all other authors providing comments. XSZ reviewed and edited the manuscript. Workload was used as the main basis to assign the authorship order among the co‐first authors.

## FUNDING INFORMATION

This work was funded by National Natural Science Foundation of China (81971726), Shanghai Jiao Tong University (YG2020YQ26 & TMSK‐2021‐148).

## CONFLICT OF INTEREST STATEMENT

The authors have declared that no conflict of interest exists.

## Supporting information


**DATA S1:** Supporting Information.

## Data Availability

The authors declare that all supporting data are available within the article. Raw data of transcriptome can be acquired from China National Center for Bioinformation (ID: PRJCA019083; https://www.cncb.ac.cn).
