# Supplementary material for: Alternating electric fields transform the intricate network of tumour vasculature into orderly parallel capillaries and enhance the anti‐angiogenesis effect of bevacizumab
Source: Cell Prolif. 2024 Aug 19;58(1):e13734. doi: 10.1111/cpr.13734 (PMC11693564; doi:10.1111/cpr.13734)
Supplement: Supplementary file 1 — DATA S1: Supporting Information. [file CPR-58-e13734-s001.docx]

**Supplementary data**

**Alternating electric fields transform the intricate network of tumor vasculature into orderly parallel capillaries and enhance the anti-angiogenesis effect of bevacizumab**

Lin Shen ^1†^, Shuai Li ^2†^, Yalin Wang ^3†^, Yi Yin ^3†^, Yiting Liu^4^, Yunlei Zhang^4, 6*^, Xuesheng Zheng ^1,2*^

^1^ Department of Neurosurgery, XinHua Hospital, Affiliated to Shanghai JiaoTong University School of Medicine; Shanghai 200092, China

^2^ Department of Neurosurgery, The Affiliated Jiangning Hospital of Nanjing Medical University; Nanjing 211199, China

^3^ School of electronic information and electrical engineering, Shanghai JiaoTong University; Shanghai 200240, China

^4^ Department of Respiratory and Critical Care Medicine, The Affiliated Jiangning Hospital of Nanjing Medical University, Nanjing Medical University; Nanjing 211100, China

^5^ The Key Laboratory of Clinical and Medical Engineering, School of Biomedical Engineering and Informatics, Nanjing Medical University; Nanjing 211100, China

* Corresponding author. E-mail: Yunlei Zhang, No. 169, Hushan Road, Nanjing 211100, China, 086+18752039836, yunleizhang@njmu.edu.cn; Xuesheng Zheng, The Affiliated Jiangning Hospital of Nanjing Medical University, Nanjing, China, xueshengzheng@gmail.com.

^†^These authors contributed equally to this work.

**Supplementary Figures:**

**
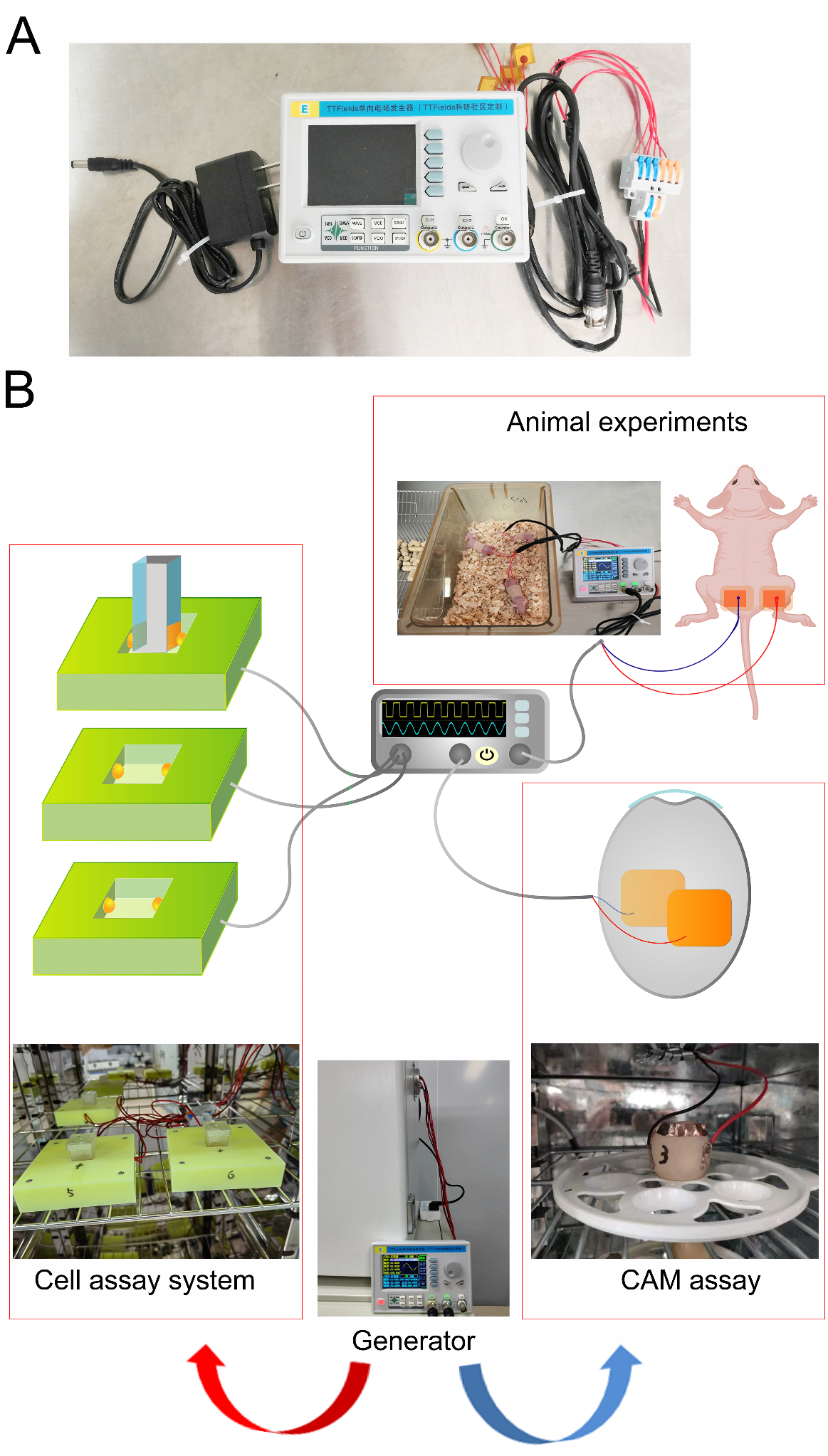
**

**Supplementary Fig. 1 A** Treatment system of alternating electric fields in this study. **B** Schematic diagram of the alternating current generator for cell-based assays, CAM assay and animal model

**
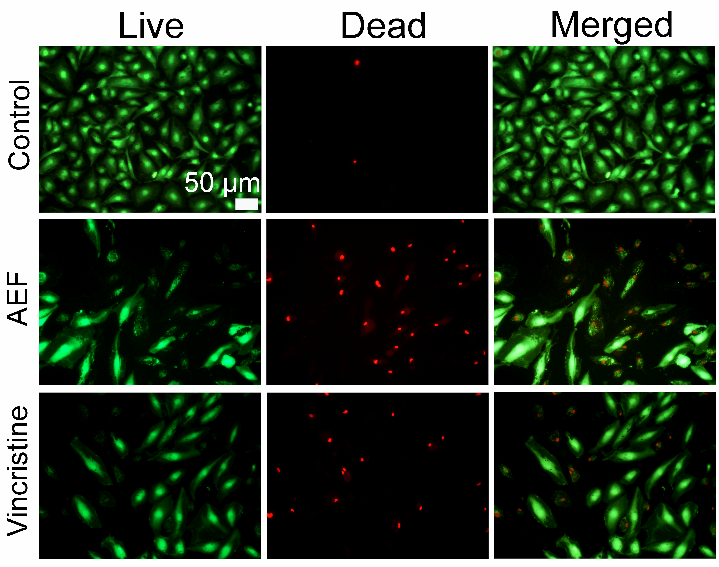
**

**Supplementary Fig. 2 AEF induces death of HUVECS.** The cells were treated by AEF or vincristine for 48 h, and then the cells were stained using calcein acetoxymethyl ester / propidium iodide mixture to mark live and dead cells. Green and red colors represent live cells (green) and dead cells (red), respectively. These images are representative of 3 independent experiments with similar results


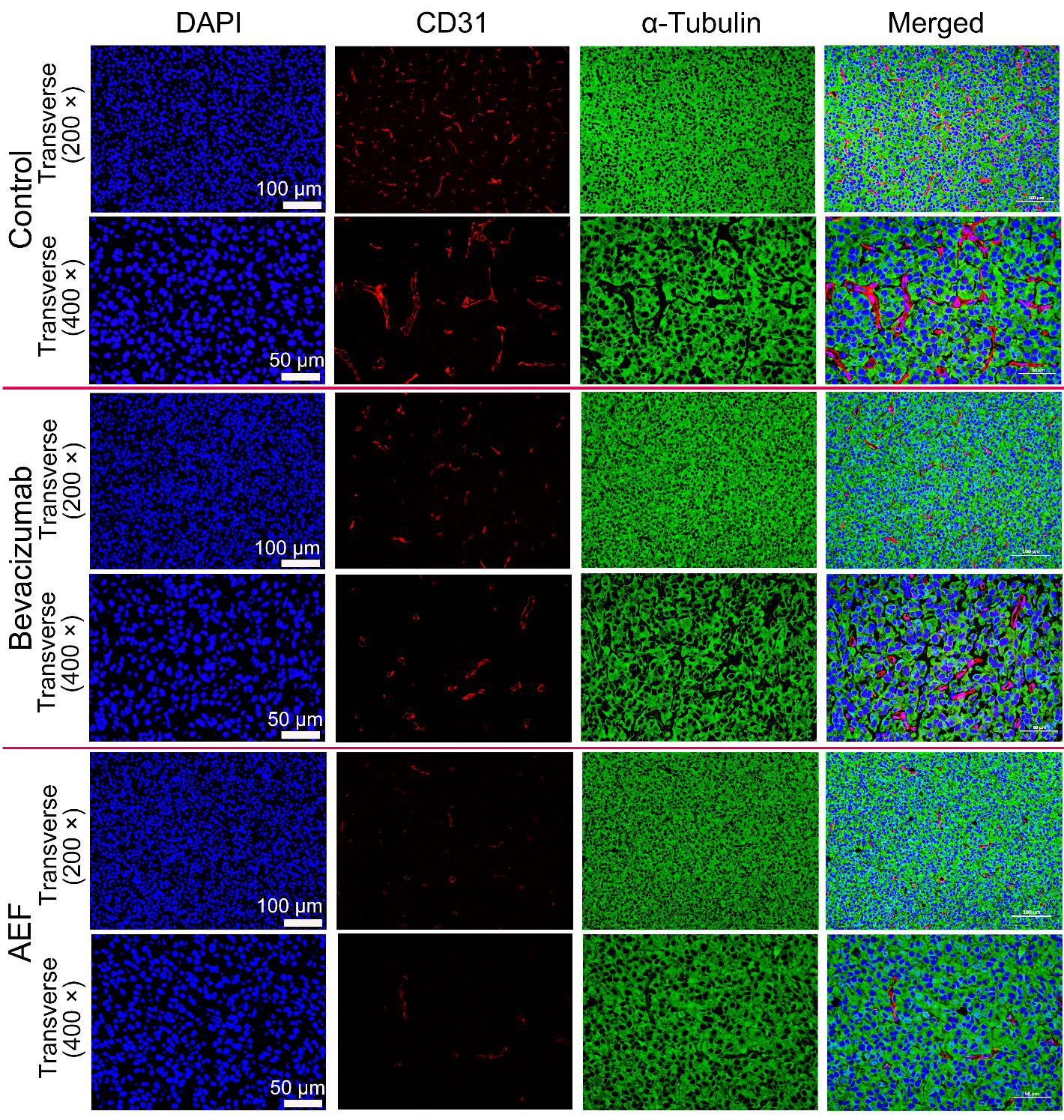


**Supplementary Fig. 3 AEF reduced angiogenesis of tumors.** The transverse orientation of tumor tissues was processed to find the CD31 labeled vessels (red color) and the α-Tubulin marked spindle (green color) through using fluorescent immunohistochemistry from Figure 4A (n = 3). These results are representative of 3 independent experiments with similar results.


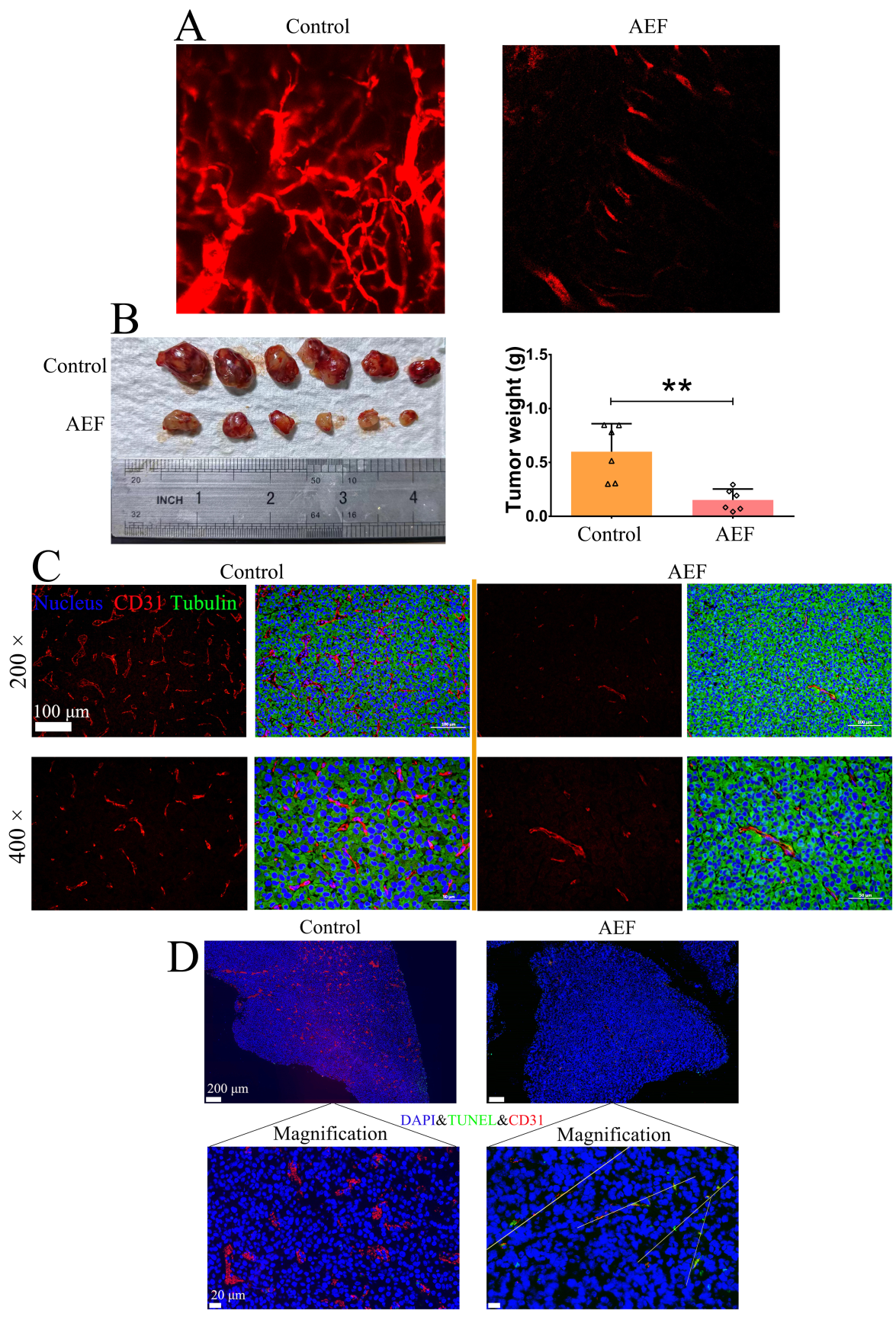


**Supplementary Fig. 4 AEF suppressed angiogenesis in MCF-7 xenograft tumors.** A In vivo tumor vasculatures imaging of the control and AEF groups, using Texas Red labeled dextran (10 mg/mL) for vessel labeling and Two-Photon Microscope for image capturing. B Tumor weight analysis of the control and AEF groups. C Fluorescent immunohistochemistry allowed for the visualization of DAPI-stained nucleus (blue color), CD31-labeled vessels (depicted in red), and α-Tubulin-marked spindles (represented in green). D A TUNEL assay was conducted to identify apoptotic cells within the tumor tissues. Apoptotic cells are visualized in green, while the vascular structures are shown in red. Apoptotic cells in the AEF group were found to overlap with the blood vessels that are oriented perpendicular to the direction of the electric field. These results are representative of 3 independent experiments with similar results.

**
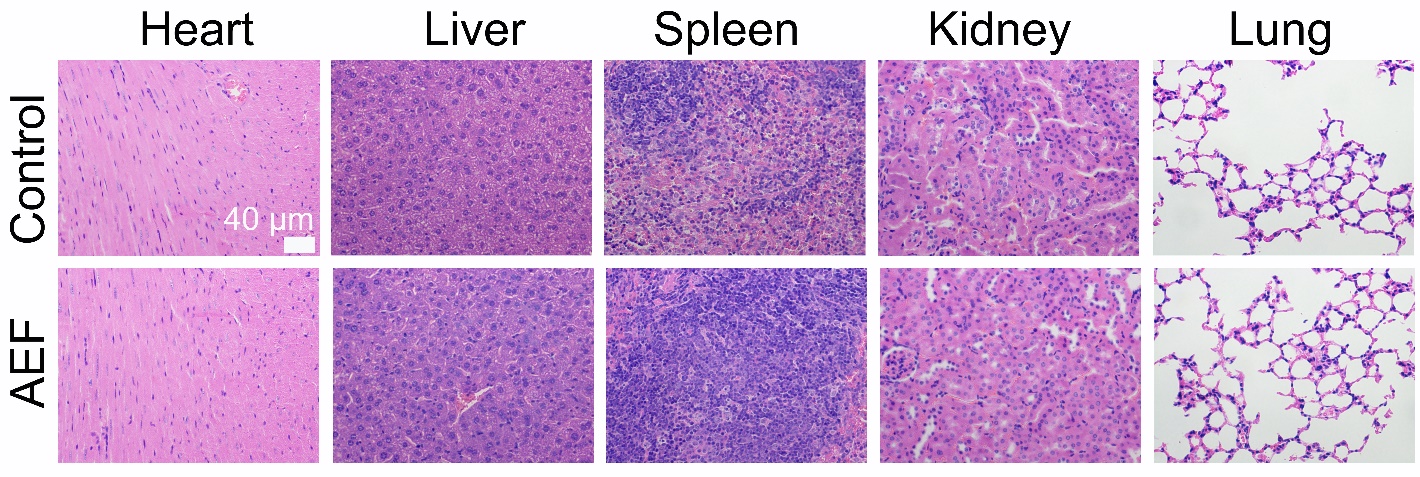
**

**Supplementary Fig. 5 H&E staining of key organs in the control and AEF groups from Fig. 4A.** These results are representative of 3 independent experiments with similar results.

**
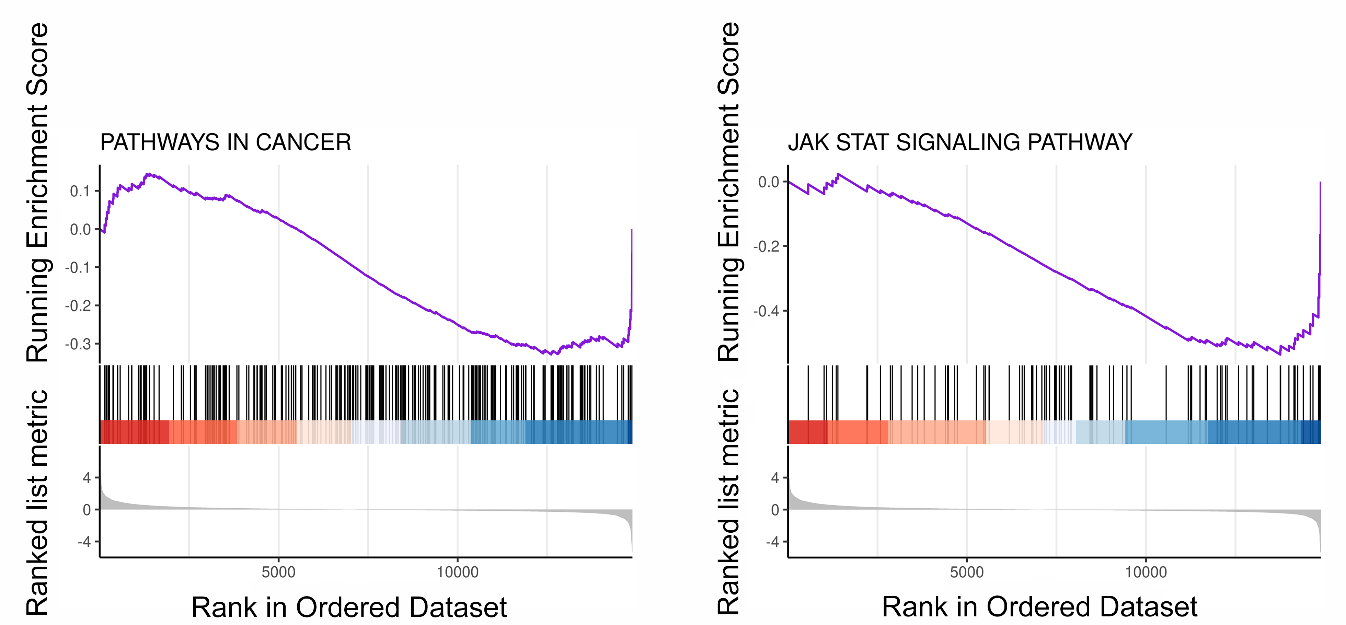
**

**Supplementary Fig. 6 Gene Set enrichment analysis (Gsea) of the control and AEF treated tumors from Fig. 4A.** These outcomes are representative of the similar findings from three independent trials. Raw data of transcriptome can be acquired from China National Center for Bioinformation (ID: PRJCA019083; <https://www.cncb.ac.cn>).

**Supplementary Table 1. Primers for Quantitative real-time PCR**

| Primer Name | Forward Sequence | Reverse Sequence |
| --- | --- | --- |
| IL-6 | AGACAGCCACTCACCTCTTCAG | TTCTGCCAGTGCCTCTTTGCTG |
| CXCL1 | AGCTTGCCTCAATCCTGCATCC | TCCTTCAGGAACAGCCACCAGT |
| CXCL2 | GGCAGAAAGCTTGTCTCAACCC | CTCCTTCAGGAACAGCCACCAA |
| CXCL3 | CCAAACCGAAGTCATAGCCAC | TGCTCCCCTTGTTCAGTATCT |
| CXCL5 | AGCTGCGTTGCGTTTGTTTAC | TGGCGAACACTTGCAGATTAC |
| CXCL8 | TTTTGCCAAGGAGTGCTAAAGA | AACCCTCTGCACCCAGTTTTC |
| MMP1 | CTCTGGAGTAATGTCACACCTCT | TGTTGGTCCACCTTTCATCTTC |
| MMP3 | CGGTTCCGCCTGTCTCAAG | CGCCAAAAGTGCCTGTCTT |
| MMP12 | CATGAACCGTGAGGATGTTGA | GCATGGGCTAGGATTCCACC |
